# Enlightening Energy Parasitism by Analysis of an ATP/ADP Transporter from Chlamydiae

**DOI:** 10.1371/journal.pbio.0050231

**Published:** 2007-08-28

**Authors:** Oliver Trentmann, Matthias Horn, Anke C. Terwisscha van Scheltinga, H. Ekkehard Neuhaus, Ilka Haferkamp

**Affiliations:** 1 Pflanzenphysiologie, Technische Universität Kaiserslautern, Kaiserslautern, Germany; 2 Department für Mikrobielle Ökologie, Universität Wien, Wien, Austria; 3 Abteilung Strukturbiologie, Max-Planck-Institute für Biophysik, Frankfurt am Main, Germany; 4 Zelluläre Physiologie/Membrantransport, Technische Universität Kaiserslautern, Kaiserslautern, Germany; Brandeis University, United States of America

## Abstract

Energy parasitism by ATP/ADP transport proteins is an essential, common feature of intracellular bacteria such as chlamydiae and rickettsiae, which are major pathogens of humans. Although several ATP/ADP transport proteins have so far been characterized, some fundamental questions regarding their function remained unaddressed. In this study, we focused on the detailed biochemical analysis of a representative ATP/ADP transporter (*Pam*NTT1), from the amoeba symbiont Protochlamydia amoebophila (UWE25) to further clarify the principle of energy exploitation. We succeeded in the purification of the first bacterial nucleotide transporter (NTT) and its functional reconstitution into artificial lipid vesicles. Reconstituted *Pam*NTT1 revealed high import velocities for ATP and an unexpected and previously unobserved stimulating effect of the luminal ADP on nucleotide import affinities. Latter preference of the nucleotide hetero-exchange is independent of the membrane potential, and therefore, *Pam*NTT1 not only structurally but also functionally differs from the well-characterized mitochondrial ADP/ATP carriers. Reconstituted *Pam*NTT1 exhibits a bidirectional orientation in lipid vesicles, but interestingly, only carriers inserted with the N-terminus directed to the proteoliposomal interior are functional. The data presented here comprehensively explain the functional basis of how the intracellular P. amoebophila manages to exploit the energy pool of its host cell effectively by using the nucleotide transporter *Pam*NTT1. This membrane protein mediates a preferred import of ATP, which is additionally stimulated by a high internal (bacterial) ADP/ATP ratio, and the orientation-dependent functionality of the transporter ensures that it is not working in a mode that is detrimental to P. amoebophila. Heterologous expression and purification of high amounts of *Pam*NTT1 provides the basis for its crystallization and detailed structure/function analyses. Furthermore, functional reconstitution of this essential chlamydial protein paves the way for high-throughput uptake studies in order to screen for specific inhibitors potentially suitable as anti-chlamydial drugs.

## Author Summary

Diverse members of the bacterial order Chlamydiales cause severe diseases in humans. Chlamydiales cannot survive and reproduce outside of host cells, due to the complete loss of several biosynthetic pathways, but rely on specialized transport systems to import the corresponding metabolites from the host. We performed a detailed biochemical analysis of a purified recombinant ATP/ADP transporter from the *Chlamydia*-related bacterium Protochlamydia amoebophila (*Pam*NTT1). Our studies revealed that *Pam*NTT1 favours ATP import into vesicles loaded with ADP, whereas the exchange of ATP in the opposite direction is negligible. Furthermore, we demonstrated that *Pam*NTT1, in contrast to the mitochondrial ADP/ATP carrier, is independent of a membrane potential. The identified biochemical characteristics of *Pam*NTT1 appear to be a sophisticated adaptation to the requirements of the intracellular lifestyle of *P. amoebophila,* serving to facilitate effective energy parasitism.

## Abstract 3

This paper explores the functional basis of how the intracellular *P. amoebophila* manages to effectively exploit the energy pool of its host cell by using the nucleotide transporter *Pam*NTT1.

## Introduction

Members of the bacterial orders Rickettsiales and Chlamydiales gained attention because they comprise several human pathogenic species causing severe diseases like typhus, pneumonia, trachoma, or sexually transmitted infections [1,2]. The obligate intracellular lifestyle of these bacteria depends upon the continuous import of a large number of metabolites from the host cell cytosol [3–8]. Consequently, both metabolism and genome size of intracellular bacteria are typically and substantially reduced compared with those of free-living bacteria [2,9–11]. For example, genome sequencing of several rickettsial and chlamydial species revealed that these bacteria show restricted nucleotide metabolism. This restriction is characterized by the inability for de novo synthesis of certain nucleotides and an impaired ability to regenerate the universal energy currency ATP [11–13]. To compensate for these limitations, specific nucleotide transport proteins (NTTs) are used, mediating either net uptake of nucleotides, the import of NAD^+^, or the counter-exchange of ATP and ADP [14–19]. The latter process has been termed “energy parasitism” and is considered fundamental for the survival of these metabolically impaired intracellular bacteria [4,20–22].

In addition to intracellular bacteria, nucleotide transport proteins have also been found in plant chloroplasts where they import cytosolic ATP under certain conditions [20]. This limited occurrence of nucleotide transport proteins in only few, largely unrelated groups of intracellular bacteria and plant plastids suggests an unusual evolutionary history for these proteins. Indeed, phylogenetic analyses showed that nucleotide transport proteins are of ancient origin (and have evolved at least 700–1,000 million years ago), and early gene duplications and lateral transfer from rickettsiae to chlamydiae or from chlamydiae to rickettsiae and (via the cyanobacterial ancestor of chloroplasts) to plants has been suggested [12,18,23–25].

The model plant Arabidopsis thaliana possesses two isoforms of plastidic ATP/ADP transporters (*At*NTT1 and *At*NTT2). *Arabidopsis* mutant plants, which lack these non-mitochondrial nucleotide transporters, are retarded in plant development and exhibit a chlorotic phenotype and spontaneous necrotic lesions under short-day conditions [26,27]. The physiological and morphological differences between the *Arabidopsis* mutant plants and the wild type were compensated by extended light conditions (long day or high light intensity) [27]. Furthermore, reduction of *ntt* transcript in potato caused remarkable effects in heterotrophic but not in autotrophic tissues (ginger shaped tubers with reduced starch contents) [28]. Latter observations underline the importance of plant NTTs in photosynthetic-inactive heterotrophic plastids and in chloroplasts during periods of reduced or missing photosynthetic activity.

Biochemical analyses of the recombinant *At*NTT1 and *At*NTT2 revealed that these transporters are highly specific for the substrates ATP and ADP. Site-directed mutagenesis of a lysine in a region that is conserved in several NTTs resulted in a markedly reduced capacity of *At*NTT1 for ATP but not for ADP import [29].

To fulfil the essential physiological role of energy exploitation, all bacterial ATP/ADP transport proteins have to catalyze the import of host-derived ATP in counter-exchange with endogenous ADP. However, one key question that has not been satisfactorily explained yet is how the required net uptake of energy into intracellular living bacteria is maintained while external ATP and ADP might compete at the binding site. This problem is even more puzzling given that all bacterial ATP/ADP transporters characterized (by heterologous expression in Escherichia coli) so far exhibit nearly similar apparent affinities and transport velocities for ATP and ADP (an overview is given in [18]). Thus, in theory, ATP/ADP transporters could also export bacterial ATP if the infected host cell exhibits a reduced energy state.

Adenine nucleotide exchange across the mitochondrial membrane is mediated by specific ADP/ATP carriers (AACs). Latter, well-characterized proteins are phylogenetically, structurally, and physiologically completely different from bacterial (and also plastidic) NTTs [30–33]. Whereas most NTTs catalyze ATP import into the bacterial cell (or into the organelle), AACs mediate export of ATP synthesized in the mitochondrion to provide cellular metabolism with energy. In this respect, a very interesting feature of mitochondrial AACs is the regulatory influence of the membrane potential on nucleotide exchange, which was analyzed in detail by the help of artificial lipid vesicles containing the purified AAC from beef heart mitochondria [34]. In the absence of a membrane potential (in “de-energized” mitochondria), ADP and ATP transport rates are nearly similar, whereas generation of a membrane potential (in “energized” mitochondria) stimulates ADP import in exchange with ATP. Whether bacterial nucleotide exchange is regulated by a similar mechanism is an interesting and still open question.

Physiological studies on native bacterial ATP/ADP transporters were hampered by the need to isolate intracellular living bacteria from eukaryotic host cells, and all attempts to cultivate metabolically stable cells outside the host failed. Because existing paralogs and possible contamination by mitochondrial ADP/ATP carriers have to be taken into account [3,4,17,19,35], and because purification of the native protein is still impossible, most NTTs characterized so far were analyzed by heterologous expression in E. coli [14,16–19,24,36,37]. However, to determine the catalytic activity of a single isoform (rather than of a mixture of carriers present in a native bacterial membrane), to study the detailed effect of a counter-exchange substrate required to drive an antiport process, and to examine the biochemistry of a carrier uncoupled from metabolic fluxes in the living bacterial cell, it is necessary to incorporate the purified protein into artificial lipid vesicles. In the current study, we established the purification procedure of the heterologously expressed ATP/ADP transporter NTT1 from P. amoebophila (*Pam*NTT1), a chlamydial endosymbiont of Acanthamoeba sp. [18,38]. This protein is an excellent and interesting candidate for further biochemical analyses, because (i) it is structurally and functionally similar and phylogenetically related to ATP/ADP transport proteins from important human pathogenic chlamydial species, (ii) it is involved in energy exploitation, and (iii) it is a recombinantly synthesized protein that is functionally inserted into the E. coli membrane at high amounts.

The purification of *Pam*NTT1 provided the basis for the functional integration into liposomes. To further clarify the mechanism of energy exploitation, special focus was laid on uptake kinetics of the reconstituted protein in the presence of internally applied ATP and ADP and also a possible influence of the membrane potential on nucleotide exchange was analyzed. Our results demonstrate that reconstitution of the purified transporter *Pam*NTT1 represents a highly sophisticated tool to gain new and detailed insights into biochemical characteristics and into orientation–function relationships of this transport protein.

## Results

### Reconstituted *Pam*NTT1 Exhibits a Preferred ATP Uptake in Counter-Exchange with ADP

To perform a detailed biochemical analysis of an ATP/ADP transporter in artificial lipid vesicles, we expressed *Pam*NTT1 heterologously in *E. coli,* purified the recombinant protein, and reconstituted the carrier into liposomes. *E. coli-*synthesized *Pam*NTT1 is a suitable source for recombinant carrier proteins, because high amounts of the heterologous transporters accumulate functionally in the bacterial membrane during synthesis [18]. Moreover, the recombinant protein was extended by an N-terminal His tag, allowing affinity purification and immunodetection.

**Figure 1:**
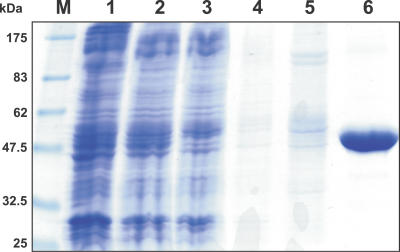
IMAC Purification of Recombinant *Pam*NTT1 Total E. coli membranes, solubilized membrane proteins and fractions from the IMAC purification steps were separated by SDS-PAGE. M**,** molecular mass marker; 1**,** total membrane proteins from *Pam*NTT1-expressing E. coli cells; 2, supernatant of DDM-solubilized membrane proteins (after centrifugation at 100,000*g*); 3, IMAC flow path; 4**,** wash step 1 (buffer medium B); 5, wash step 2 (buffer medium W); 6, eluate (buffer medium E).

Treatment of the E. coli membrane fraction with the detergent *N*-dodecyl-β-maltoside (DDM) led to efficient solubilization of a majority of the membrane proteins (Figure 1, lane 2), including the recombinant *Pam*NTT1. SDS-PAGE confirmed that *Pam*NTT1 was purified to apparent homogeneity using immobilized metal affinity chromatography (IMAC) (Figure 1, lanes 3–6). By this method, it was possible to purify about 0.75 mg *Pam*NTT1 from 1 l E. coli culture harvested 4 h after induction.

**Figure 2:**
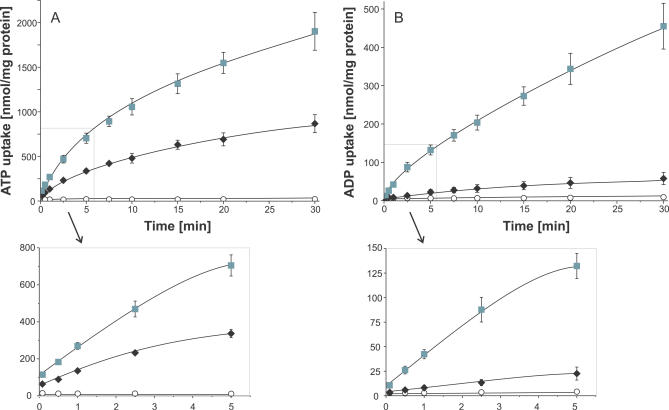
Time Dependency of Recombinant *Pam*NTT1 Mediated Uptake of Nucleotides into Proteoliposomes Proteoliposomes were incubated with 50 μM [α^32^P] labelled ATP or ADP for the indicated time. Concentration of loaded nucleotides was 10 mM. Uptake was stopped by separation of external nucleotides from proteoliposomes by anion exchange chromatography. (A) Time dependence of ATP uptake into ADP-loaded (gray squares), ATP-loaded (black diamonds), and unloaded proteoliposomes (open circles). (B) Time dependence of ADP uptake into ADP-loaded (gray squares), ATP-loaded (black diamonds), and unloaded proteoliposomes (open circles). Data are the means of at least three independent experiments, each with four technical replicates. An expanded time scale for the early time points (0 to 5 min) of the linear rate is given below the corresponding uptake kinetic.

To analyze the functionality of the purified transporter and to ascertain the time-linear phase of uptake, proteoliposomes were incubated in buffer medium containing radioactively labelled [α^32^P] ATP or [α^32^P] ADP. Reconstituted *Pam*NTT1 mediated nucleotide uptake exclusively into vesicles containing ADP or ATP, whereas no measurable luminal accumulation of radioactivity occurred in unloaded proteoliposomes (control) (Figure 2A and 2B) or into loaded or unloaded liposomes lacking reconstituted *Pam*NTT1 (unpublished data). *Pam*NTT1-mediated import of ATP, as well as ADP, into loaded vesicles was linear with time for at least 1 min of incubation (Figure 2A and 2B). It appears remarkable that ATP uptake into proteoliposomes was always substantially higher than ADP uptake (Figure 2A and 2B), because this contrasts with previous findings obtained in uptake studies of recombinant *Pam*NTT1 using the heterologous host E. coli [18].

Interestingly, the data revealed an unexpected influence of the type of loaded substrate (present at the liposomal interior) on nucleotide import. In general, ATP import (ATP_im_) in exchange with internal ADP (ADP_ex_) (ATP_im_/ADP_ex_) was about two times faster than ATP import into ATP-loaded vesicles (ATP_im_/ATP_ex_), and ADP homo-exchange (ADP_im_/ADP_ex_) was about ten times faster than ADP import in counter-exchange with ATP (ADP_im_/ATP_ex_) (Figure 2A and 2B).

ATP_im_/ADP_ex_ was rapid and nearly equilibrated at 30 min (at about 2,000 nmol/mg protein). Compared with the ATP_im_/ADP_ex_ hetero-exchange, ATP_im_/ATP_ex_ homo-exchange was slower, and the rate declined progressively until 20–30 min, when no further substantial accumulation of radioactivity was detectable (at about 700 nmol/mg protein) (Figure 2A). *Pam*NTT1-mediated ADP_im_/ADP_ex_ homo-exchange was slower than ATP_im_/ATP_ex_ homo-exchange and obviously approached equilibration after about 30 min. Remarkably, ADP_im_/ATP_ex_ hetero-exchange was only slightly stimulated when compared with the corresponding control (Figure 2B). ADP_im_/ATP_ex_ was equilibrated at about 20 min (at only 60 nmol/mg protein) and appeared (in the linear phase of uptake) at least 20 times slower than in the reciprocal transport mode (ATP_im_/ADP_ex_) (Figure 2A and 2B).

To further characterize the function of substrates present at the liposomal interior on nucleotide import, additional uptake studies were performed at different luminal ADP/ATP ratios (10 mM final nucleotide concentration; nucleotide import into ATP-loaded liposomes was set to 1). Increasing ADP/ATP ratios in the proteoliposomes resulted in higher ATP uptake rates with a 2.34-times increase for the exclusive presence of ADP (Table 1). As described above, the nature of the luminal adenine nucleotide exhibited an even more noticeable effect on the relative ADP import affinities. Accordingly, a substantial increase of the ADP uptake rate becomes obvious in dependence on rising luminal ADP/ATP ratios, sole luminal ADP led to a 12-times increased *Pam*NTT1-mediated ADP uptake (Table 1).

**Table 1:**
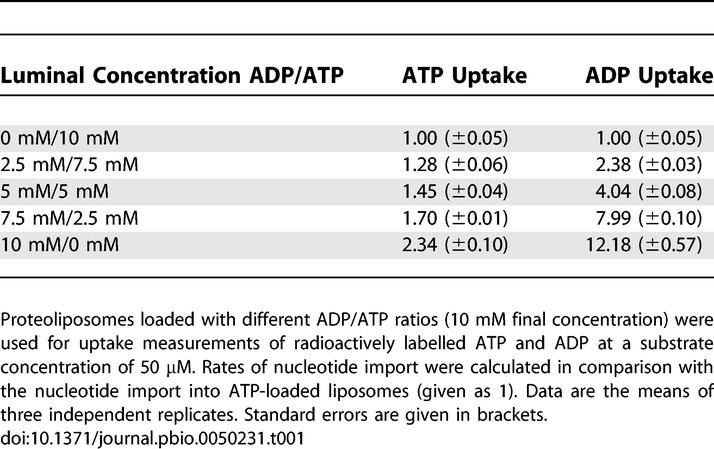
Influence of Rising ADP/ATP Ratios in the Loaded Substrate on Nucleotide Import

**Figure 3:**
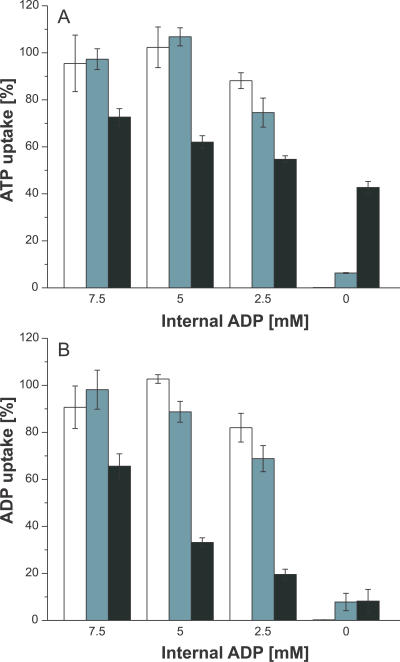
Influence of Substrates Present at the Liposomal Interior on Nucleotide Import ATP import (A) and ADP import (B) into liposomes loaded with decreasing internal ADP concentrations in absence of additional nucleotides (white bars) and in presence of GTP (light gray bars) or ATP (dark gray bars) was measured. Nucleotide uptake into liposomes loaded with solely 10 mM ADP was set to 100% (unpublished data) and the transport into the differently loaded liposomes was calculated accordingly. Data represent net values calculated by subtraction of nucleotide import into unloaded liposomes without reconstituted protein. Data are the means of three independent experiments.

To analyze whether the lack of the favoured counter exchange substrate ADP or the presence of ATP at the proteoliposomal interior caused the lowered nucleotide import capacity, the accumulation of radioactivity into liposomes loaded with different concentrations of ADP in the absence of any additional nucleotide (ADP-NTP) or in the presence of the nonsubstrate GTP (ADP+GTP) was determined (Figure 3). The resulting data were normalized to the import into liposomes loaded with 10 mM ADP (=100%). In general, both loading conditions (ADP-NTP and ADP+GTP) led to nearly comparable uptake rates (Figure 3). Decrease of the internal ADP concentration to about 5 mM caused no significant reduction of nucleotide uptake into ADP-NTP– and ADP+GTP–loaded liposomes when compared to the corresponding import into proteoliposomes loaded with 10 mM ADP (100%). However, at an interior concentration of 2.5 mM ADP, a slight reduction of the nucleotide-import capacity of ADP-NTP– and ADP+GTP–loaded proteoliposomes was observed, probably caused by the limitation of counter-exchange substrate (Figure 3). Mixed ADP+ATP–loaded liposomes led to lower uptake rates when compared with the corresponding import into solely ADP- or ADP+GTP–loaded vesicles, and decreasing internal ADP/ATP ratios reduced ADP uptake (Figure 3B) to a higher extent than did ATP uptake (Figure 3A). Because sole presence of GTP at the proteoliposomal interior caused slight nucleotide uptake, we assume that either GTP contains minor contaminations with adenine nucleotides or that very high concentrations of GTP may also function as substrate of the *Pam*NTT1.

Our results demonstrate that reduction of import into the differently ADP+ATP–loaded liposomes is rather caused by the presence of the additional export substrate ATP than by a limitation of the counter-exchange substrate ADP.

**Figure 4:**
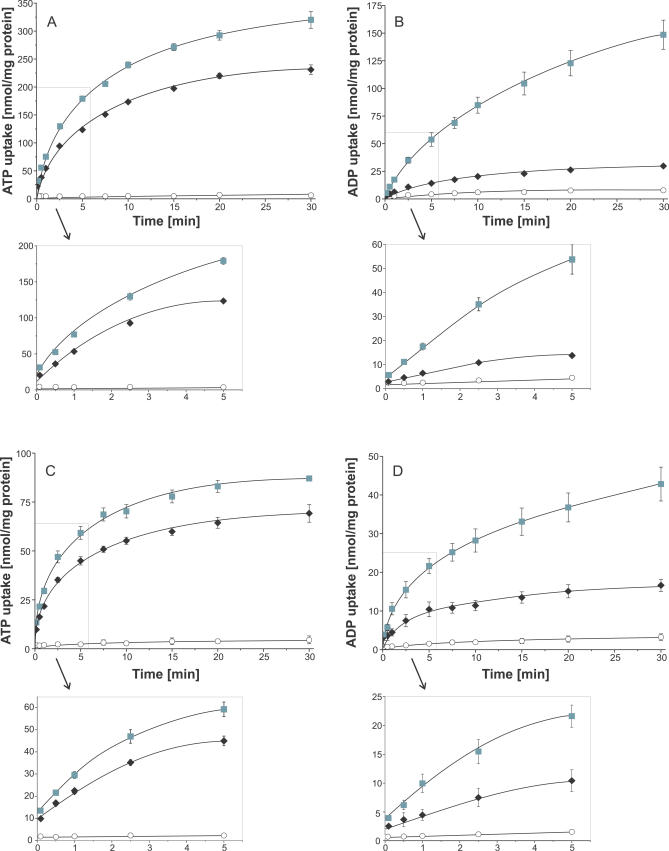
Time Dependency of *Pam*NTT1-Mediated Nucleotide Uptake into Proteoliposomes Loaded with Different Nucleotide Concentrations Proteoliposomes were incubated with 100 μM (A and B) or 50 μM (C and D) [α^32^P]-labelled ATP or ADP for the indicated time. Concentration of loaded nucleotides was 1 mM (A and B) or 50 μM (C and D), respectively. Uptake was stopped by separation of external nucleotides from proteoliposomes by anion exchange chromatography. (A and C) Time dependence of ATP uptake into ADP loaded (gray squares), ATP loaded (black diamonds), and unloaded proteoliposomes (open circles). (B and D) Time dependence of ADP uptake into ADP-loaded (gray squares), ATP-loaded (black triangles), and unloaded proteoliposomes (open circles). Data are the means of at least three independent experiments. An expanded time scale for the early time points (0 to 5 min) of the linear rate is given below the corresponding uptake kinetic.

In most previous experiments, substrates were internally applied at high concentrations (2.5 to 10 mM), and the externally nucleotides were present at significantly lower concentrations (50- to 200-fold), which results in a nucleotide gradient across the liposomal membrane. To analyze whether this gradient causes the observed preference of ATP_im_/ADP_ex_ exchange and the low rates of the ADP_im_/ATP_ex_ transport, we analyzed nucleotide uptake into liposomes in the presence of a low (Figure 4A and 4B), and in the absence of a nucleotide gradient (Figure 4C and 4D). In general, nucleotide transport was slower and equilibrium was reached faster in proteoliposomes with a reduced nucleotide gradient (Figure 4) when compared with uptake measurements under saturating interior nucleotide concentrations (Figure 2). This is due to limiting counter-exchange substrate concentrations at the proteoliposomal interior. For example, 100 μM ATP_im_/1 mM ADP_ex_ transport was equilibrated after 20 to 30 min at about 300 nmol/mg protein (Figure 4A), and 50 μM ATP_im_/50 μM ADP_ex_ transport was equilibrated after 15 min at about 80 nmol/mg protein (Figure 4C). In the linear time phase, *Pam*NTT1 exhibited about 1.5 times higher transport rates for ATP_im_/ADP_ex_, when compared with the ATP homo-exchange, and ADP homo-exchange is at least two times higher than the ADP_im_/ATP_ex_ transport (Figures 2 and 4). In accordance with the results obtained under saturating interior nucleotide concentrations (Figure 2), *Pam*NTT1 prefers ATP_im_/ADP_ex_ exchange and suppresses the opposite direction of transport, and therefore, this feature is independent of the nucleotide gradient present at the vesicle membrane.

### *Pam*NTT1-Mediated Nucleotide Exchange Is Independent of the Membrane Potential

It is well known that transport characteristics of mitochondrial ADP/ATP carriers are regulated by the membrane potential [34]. To analyze whether the potassium gradient in the applied system causes the observed preferred ATP_im_/ADP_ex_ transport, we determined transport activity of reconstituted *Pam*NTT1 in the presence of different potassium concentrations at the liposomal interior or exterior. The ratios of exchange were unaffected by the applied buffer media (unpublished data).

**Figure 5:**
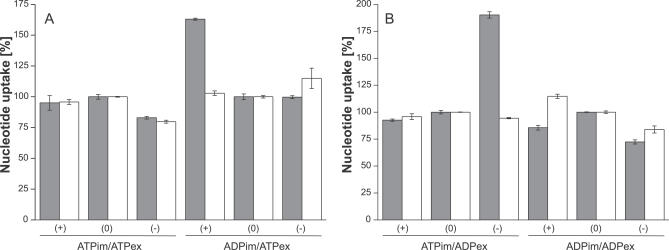
Influence of the Membrane Potential on *Pam*NTT1 and Mitochondrial AAC-Mediated Uptake Reconstituted recombinant *Pam*NTT1 or reconstituted mitochondrial total membrane proteins were incubated with 50 μM [α^32^P]-labelled ADP or ATP. Concentration of internal ATP (A) or ADP (B) was 10 mM. For transport measurements in absence of a membrane potential (0) similar buffer media were present at the proteoliposomal exterior and interior. Generation of a membrane potential was achieved by addition of 20 μM of valinomycin to proteoliposomes with different potassium concentrations at the interior and exterior. According to the flux of the applied potassium concentrations, the corresponding membrane potential was calculated. Potassium influx resulted in a theoretical external negative membrane potential (**−**) of **−**40 mM, and potassium efflux in a theoretical external positive membrane potential (**+**) of +40 mV. The exchange rate is given in % normalized to the transport in absence of a membrane potential (100%). Data of AAC-mediated (gray bars) and NTT-mediated (white bars) nucleotide exchange are the means of three independent experiments.

We also compared the exchange rates of *Pam*NTT1 and of mitochondrial AAC from yeast in the absence and the presence of a membrane potential. To minimize a possible leakage of potassium across the proteoliposomal membrane, which would result in the generation of a membrane potential, the same buffer media were applied at the interior and exterior side of the proteoliposome (30 mM potassium gluconate, 100 mM tricine, pH 7.5). Hetero- and homo-exchange of ATP and ADP in the absence of a potassium gradient were measured and set to 100% (Figure 5). A membrane potential was generated by the application of the ionophore valinomycin. The direction and extent of the potential depends on the difference in the potassium concentrations at the interior and exterior side of the membrane. For the liposomes applied in this study, an external positive (theoretical) membrane potential of about +40 mV (30 mM K^+^ external/150 mM K^+^ internal: potassium efflux) and an external (theoretical) negative membrane potential of −40 mV (150 mM K^+^ external/30 mM K^+^ internal: potassium influx) were calculated, respectively. Transport measurements revealed that neither a positive nor a negative membrane potential substantially influenced nucleotide exchange of *Pam*NTT1, which was purified and reconstituted in presence of DDM (Figure 5) or Triton (Triton-X-100) (unpublished data). However, mitochondrial AAC-mediated ADP_im_/ATP_ex_ transport was stimulated (to about 160%) by an external positive membrane potential, and ATP_im_/ADP_ex_ exchange was stimulated (to about 190%) by an external negative membrane potential (Figure 5). Our results demonstrate that in contrast to mitochondrial adenine nucleotide exchange, *Pam*NTT1-mediated transport is independent of a membrane potential.

### Affinities, but Not Maximal Velocities of *Pam*NTT1, Are Considerably Influenced by the Type of Counter-Exchange Nucleotide

To analyze in more detail the effect of the export substrate ADP on *Pam*NTT1-mediated nucleotide import, we determined the kinetic parameters of this protein in ATP-, mixed (ATP+ADP)–, or ADP- (10 mM) loaded proteoliposomes. Rising concentrations of exogenously supplied ATP led to increased rates of nucleotide uptake, reaching saturation at about 23,000 nmol (mg protein)^−1^ h^−1^ in the differently loaded proteoliposomes (Table 2). In presence of varied internal ADP/ATP ratios, the maximal transport velocities for ADP uptake (of about 6,000 to 8,000 nmol (mg protein)^ −1^ h^−1^) resembled the maximal velocities of the import into solely ADP- or ATP-loaded liposomes (Table 2). Interestingly, estimation of the apparent Michaelis-Menten constant (*K*_M_) values showed that *Pam*NTT1 exhibited different substrate affinities depending on the type of counter-exchange nucleotide (Table 2). At a substrate concentration of 17 μM, *Pam*NTT1 exhibited a half-maximal velocity of ATP_im_/ADP_ex_, whereas a six-times-higher ATP concentration was required when solely ATP was internally present. Furthermore, the affinity of *Pam*NTT1 for external ADP was at least ten times higher in the homo-exchange mode of transport (ADP_im_/ADP_ex_) when compared with the hetero-exchange mode (ADP_im_/ATP_ex_) (≥918 μM). Approximately equivalent *K*_M_ values were estimated for the ADP_im_/ADP_ex_ and ATP_im_/ATP_ex_ homo-exchange mode of transport (Table 2). Analyses of kinetic parameters of ATP or ADP import into mixed (ATP+ADP)–loaded liposomes revealed intermediate affinities when compared with solely ADP- or ATP- loaded liposomes. Decreasing internal ATP/ADP ratios led to overall enhanced affinities of *Pam*NTT1 for nucleotide import. The determination of kinetic parameters for ADP uptake was problematic in the presence of high internal ATP concentrations, and the resulting *K*_M_ values are therefore possibly underestimated. Taken together, these data demonstrate that *Pam*NTT1 imports ATP at highest maximal velocity (*V*_max_) independent of the loaded nucleotide and at highest affinity only if ADP, but not ATP, is present at the proteoliposomal interior.

**Table 2:**
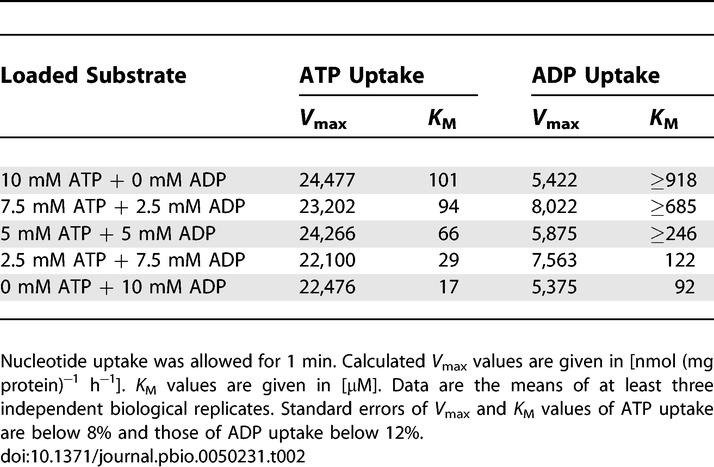
*K*_M_ Values and *V*_max_ Values of ATP and ADP Import into Loaded *Pam*NTT1 Proteoliposomes

### Is the ATP Import Defying all Competition?

**Figure 6:**
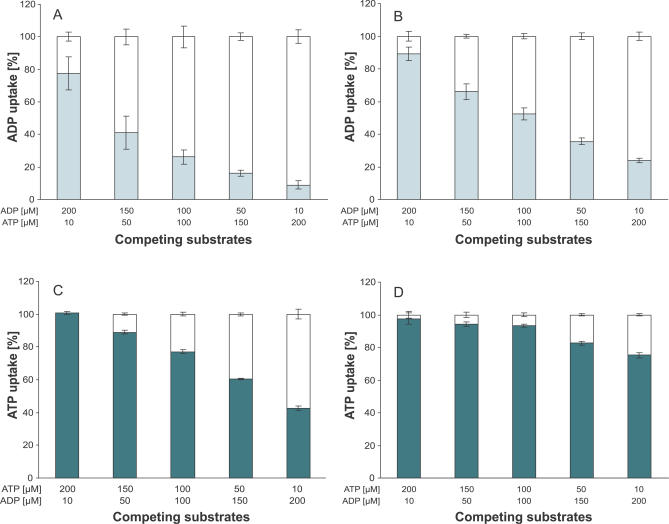
Influence of Varying External ATP/ADP Ratios on [α^32^P]-Labelled Nucleotide Import into ATP (A and D) and ADP (B and C) Loaded Liposomes Concentration of the labelled nucleotide is given in the upper line of the *x*-axis legend. [α^32^P]–labelled nucleotide import in absence of competing substrate is given as 100% (white bars). Uptake in the presence of additional competing substrate (concentration given in the bottom line of the *x*-axis legend) is normalized to the corresponding transport in absence of competing substrate (100%). [α^32^P] ADP uptake in the presence of competing ATP (light gray bars) into ATP loaded (A) or ADP loaded (B) proteoliposomes. [α^32^P] ATP uptake in presence of competing ADP (dark gray bars) into ADP-loaded (C) or ATP-loaded (D) proteoliposomes. For all tested exchanges, nucleotide import was stopped in the time linear phase of uptake. Data are the means of three independent experiments and represent net values calculated by subtraction of the corresponding transport into unloaded proteoliposomes.

Because of the high affinity and maximal velocity, the ATP_im_/ADP_ex_ seems to be the favoured exchange. To analyze whether ADP under certain conditions can compete with ATP as an import substrate of *Pam*NTT1, the effect of simultaneous presence of different exterior ATP and ADP concentrations on transport into ATP- and ADP-loaded proteoliposomes was determined. Uptake of labelled nucleotide was performed in the absence (set to 100%) and presence of a nonlabelled competing substrate (normalized to the corresponding 100% value). Our data demonstrate that the already-lowest concentrations of nonlabelled ATP effectively reduced [α^32^P] ADP import into ATP-loaded liposomes (Figure 6A). The presence of 50 μM ATP (1/3 of the exterior ADP concentration) led to a significant inhibition of the 150 μM [α^32^P] ADP_im_/ATP_ex_ to about 40% (Figure 6A) and of the 150 μM [α^32^P] ADP_im_/ADP_ex_ to about 66% (Figure 6B) when compared to the corresponding nonaffected ADP import. Twenty-fold excess of nonlabelled ATP reduced the import of 10 μM [α^32^P] ADP into ATP- and ADP-loaded liposomes to a value of about 8% (Figure 6A) and 20% (Figure 6B), respectively. Although the inhibition pattern of nonlabelled ADP on [α^32^P] ATP_im_/ADP_ex_ (Figure 6C) resembles the influence of nonlabelled ATP on [α^32^P] ADP_im_/ADP_ex_ (Figure 6B) the rate of ADP import-inhibition by competing ATP is generally higher (Figure 6B and 6C). The recombinant *Pam*NTT1 exhibits lowest affinities and a low maximal velocity for ADP_im_/ATP_ex_ (Table 2). Therefore, it is not surprising that ATP can most effectively compete with ADP for import into ATP-loaded liposomes (Figure 6A) and that a 20-fold excess of ADP caused only a slight reduction (to about 75%) of the second-best [α^32^P] ATP_im_/ATP_ex_ transport (Figure 6D). These data demonstrate that in accordance with the determined kinetic parameters, lowest concentrations of ATP (1/3 of the ADP concentration) are sufficient to effectively displace the import substrate ADP (Figure 6A) and that high concentrations of ADP are unable to significantly suppress [α^32^P] ATP import in counter-exchange with ATP (Figure 6D).

### *Pam*NTT1 Exhibits a Bidirectional Orientation in Proteoliposomes Independent of the Luminal Counter-Exchange Substrate

The foregoing data demonstrate that *Pam*NTT1 imports ATP with the highest velocity and exhibits the highest nucleotide ATP import affinity when ADP is present at the proteoliposomal interior. In contrast, *Pam*NTT1 exhibits a low import velocity and a very low import affinity when ADP is present at the exterior and ATP at the opposite side. At first glance, this effect is hard to understand if *Pam*NTT1 is inserted in a bidirectional (nearly 1:1) orientation into the liposome. To examine whether the presence of either ATP or ADP during preparation of proteoliposomes might affect the insertion of *Pam*NTT1 into the vesicle, we analyzed the efficiency of reconstitution and the orientation of *Pam*NTT1 in differently loaded liposomes by protease treatment, SDS-PAGE, and immunostaining.

**Figure 7:**
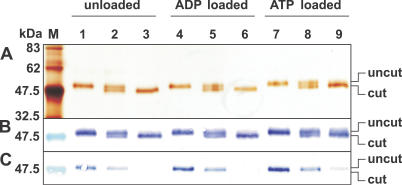
Factor Xa Digestion of Reconstituted *Pam*NTT1 After 8 h of incubation at 23 °C, protease activity was stopped by addition of PMSF (1 mM). (A) Silver-stained SDS-PAGE. (B) Western blot immuno-decorated with a *Pam*NTT1-specific serum. (C) Western blot immuno-decorated with a monoclonal anti–poly His IgG. M, molecular mass marker; 1, 4, 7, proteoliposomes without protease treatment; 2, 5, 8, protease treated proteoliposomes; 3, 6, 9, protease treated and solubilized (2% DDM) proteoliposomes. Loaded nucleotides were always present at concentrations of 10 mM. SDS-PAGE and Western blot analyses represent a typical result as observed in seven independent experiments.

Silver staining and immunodetection with an antiserum raised against purified *Pam*NTT1 (Figure 7A and 7B; lanes 1, 4, 7) or with a His tag–specific antibody (Figure 7C; lanes 1, 4, 7) revealed that the present single band corresponds to the recombinant *Pam*NTT1 and that the amount of reconstituted protein is comparable in differently loaded liposomes.

To ascertain the orientation of *Pam*NTT1 in the vesicles, unloaded or ADP- or ATP-loaded proteoliposomes were incubated with factor Xa. Proteolysis of the recombinant *Pam*NTT1 by factor Xa resulted in the removal of a 2.4-kDa peptide containing the His tag (Figure 7). After factor Xa treatment, the ratio of uncut to cut protein is comparable in the differently loaded liposomes (Figure 7A–7C; lanes 2, 5, 8). Proteolysis of reconstituted *Pam*NTT1 was only partial even over prolonged incubation (unpublished data). However, destruction of the proteoliposomal integrity by the application of a high detergent concentration (2% DDM) resulted in an almost complete cleavage of the N-terminal His tag (Figure 7A–7C; lanes 3, 6, 9).

Thus, a (by eye) estimated amount of about 50% of the reconstituted *Pam*NTT1 protein was not accessible to factor Xa and therefore obviously exhibited an orientation with the His tag directed to the lumen of the proteoliposome. These results demonstrated that both the efficiency and the direction of *Pam*NTT1 insertion into the vesicle were not influenced by the type of counter-exchange substrate present during preparation of the proteoliposomes.

### Only *Pam*NTT1 Inserted into the Liposome with the N Terminus Exposed to the Lumen Is Functional

The preferred ATP_im_/ADP_ex_ transport and the marginal transport rates of the opposite exchange (ADP_im_/ATP_ex_) seem to argue against a bidirectional (nearly 1:1) orientation of *Pam*NTT1 in proteoliposomes. The observed (directed) transport activity of reconstituted *Pam*NTT1 in liposomes can be explained if we postulate that only one mode of orientation exhibits total catalytic activity.

**Figure 8:**
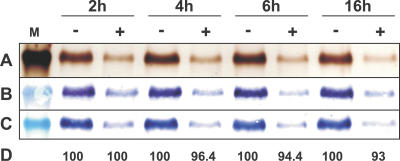
Asp-N Digestion of Reconstituted *Pam*NTT1 After 2, 4, 6, and 16 h of incubation at 37 °C, EDTA (10 mM) was added to untreated (**−**) or Asp-N treated (+), ADP-loaded (10 mM) proteoliposomes to stop protease activity. The indicated marker lane (M) corresponds to a molecular mass of 47.5 kDa. (A) Silver stained SDS-PAGE. (B) Western blot immuno-decorated with a *Pam*NTT1 specific serum. (C) Western blot immuno-decorated with a monoclonal anti–poly His IgG. (D) Uptake of ATP into protease treated proteoliposomes is given in percent corresponding to the activity of untreated proteoliposomes. Uptake assays were carried out in the presence of 50 μM [α^32^P] ATP for 5 min. Transport measurements are the means of three independent experiments, each with four technical replicates. The given data represent the net nucleotide import calculated by subtraction of the control (uptake into treated or untreated unloaded proteoliposomes). SDS-PAGE and Western blot analyzes represent a typical result as observed in five independent experiments.

**Figure 9:**
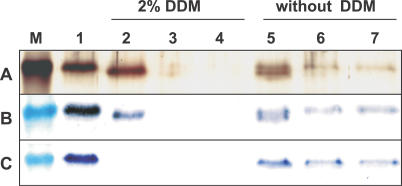
Combined Factor Xa and Asp-N Digestion of Reconstituted *Pam*NTT1 Initially intact or DDM solubilized ADP-loaded proteoliposomes were treated with factor Xa for 8 h at 23 °C followed by Asp-N digestion carried out at 37 °C. Factor Xa activity was stopped with PMSF (1 mM) and Asp-N activity was stopped with EDTA (10 mM). (A) Silver-stained SDS-PAGE. (B) Western blot immuno-decorated with a *Pam*NTT1 specific serum. (C) Western blot immuno-decorated with a monoclonal anti poly His IgG. The indicated marker lane (M) corresponds to a molecular mass of 47.5 kDa; 1, untreated proteoliposomes; 2, 5, factor Xa treated proteoliposomes; 3, 6, factor Xa treated proteoliposomes followed by Asp-N treatment for 2 h (3, 6); and 4 h (4, 7), respectively.

**Figure 10:**
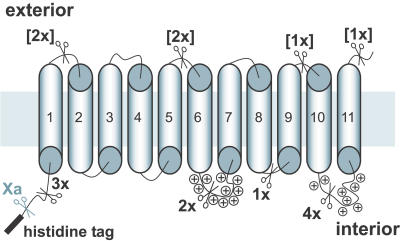
Simplified Topology Model of *Pam*NTT1 The functionally integrated transporter exposes the N-terminal His tag to the lumen. Eleven transmembrane domains in *Pam*NTT1 have been predicted according to TMpred (http://www.ch.embnet.org). The unique factor Xa cleavage site and the 16 Asp-N cleavage sites are indicated by scissor symbols. To distinguish exterior Asp-N cleavage sites, which are not accessible in the functional fraction of *Pam*NTT1, the corresponding numbers are given in brackets. Accumulation of positively charged residues is marked (plus signs).

To gain additional insights into orientation/function relationships of the reconstituted *Pam*NTT1, we analyzed the influence of endoprotease Asp-N treatment on the transport rate of the reconstituted transport protein (Figures 8 and 9). Proteolysis of ADP-loaded proteoliposomes for 2 h resulted in the cleavage of the majority of reconstituted *Pam*NTT1 (Figure 8A–8C; 2 h). Although, the amino acid sequence of *Pam*NTT1 exhibits 16 potential cleavage sites located in different hydrophilic regions of the protein (Figure 10), a fraction of *Pam*NTT1 obviously persisted protease treatment for at least 16 h (Figure 8A–8C). To clarify whether the Asp-N–protected or the cleaved portion of reconstituted *Pam*NTT1 corresponds to the functional protein, we determined the import capacity of protease treated ADP-loaded (and unloaded) proteoliposomes by transport measurements. Interestingly, no decrease of ATP transport was observed after 2 h of proteolysis, and even an extended incubation with Asp-N resulted only in a slight reduction of ATP uptake (maximal 7%) when compared with the control (ATP import into untreated proteoliposomes) (Figure 8D). An accumulation of radioactively labelled ATP caused by a possible leakage of the liposomes due to protease treatment was ruled out, because Asp-N–treated as well as untreated unloaded proteoliposomes exhibited comparable, low background values (less than 8 % of ATP_im_/ADP_ex_). This analysis demonstrates that only a minor fraction of totally reconstituted *Pam*NTT1 mediates nucleotide transport and that this functional protein is protected against Asp-N digestion.

Consequently, it was necessary to determine the orientation of the Asp-N–protected, functional *Pam*NTT1 proteins in liposomes. Therefore, we subsequently incubated reconstituted *Pam*NTT1 with the endoproteases factor Xa and Asp-N, and we analyzed proteolysis of the carrier protein by SDS-PAGE and immunostaining. Treatment of the proteoliposomes with factor Xa allowed us to distinguish between the two orientations of the protein, and the subsequent use of Asp-N protease resulted in the identification of the functional protein. Thus, this approach is suitable to determine the orientation of the functional fraction of *Pam*NTT1.

*Pam*NTT1 extended by the His tag exhibited a single band at about 47.5 kDa (Figure 9; lane 1). In the presence of a high detergent concentration, both proteases completely digested *Pam*NTT1 (Figure 9; lanes 2–4). Incubation of intact proteoliposomes with factor Xa resulted in the cleavage of the N terminus in about 50% of the reconstituted protein (Figure 9; lane 5). Factor Xa–treated liposomes were subsequently digested with protease Asp-N for 2 and 4 h, (Figure 9; lanes 6 and7). The resulting single band was still detectable by the His tag–specific antibody (Figure 9C; lanes 6 and 7); it exhibited a molecular weight of about 47.5 kDa and therefore corresponds to the undigested *Pam*NTT1, whereas the truncated *Pam*NTT1 (lacking the His tag) was completely fragmented (Figure 9B; lanes 6 and 7). These experiments demonstrate that the functional portion of *Pam*NTT1 is Asp-N resistant (Figure 8). Moreover, the insensitivity of functional *Pam*NTT1 towards factor Xa (Figure 9) implies that the N terminus of this fraction is directed into the proteoliposomal lumen.

## Discussion

Chlamydial symbionts of free-living amoebae, such as *P. amoebophila,* exhibit remarkable similarities to human pathogenic Chlamydiaceae, including Chlamydia trachomatis or *Chlamydophila pneumoniae,* and thus can serve as model systems for the analysis of the interaction between intracellular bacteria and their host cells [39]. An essential common feature of both groups, and also of the parasitic Rickettsiales, is the capacity to perform nucleotide import and energy parasitism by specialized NTTs [14,17–19,24]. In contrast to all other bacterial ATP/ADP transporters analyzed so far, the recombinant homolog from P. amoebophila (*Pam*NTT1) investigated in this study meets the requirements for a detailed biochemical characterization, especially because purified *Pam*NTT1 (Figure 1) exhibited an extraordinarily high specific activity.

The reconstituted carrier showed significant import of ATP or ADP into liposomes loaded with counter-exchange substrates (ADP or ATP), whereas transport was absent in unloaded proteoliposomes (Figures 2 and 4). This provides evidence for the strict coupling of nucleotide import and export and confirms the previously described counter-exchange mode of *Pam*NTT1 [18].

Although *Pam*NTT1 exhibited a favoured import of ATP in exchange with ADP and moderate transport rates for the ATP and ADP homo-exchange, the import of ADP in exchange with ATP was only slightly in excess of the background, observed in unloaded liposomes. The preference of the ATP import in counter-exchange with ADP and the low activity of the opposite hetero-exchange were independent of the detergent applied during purification and reconstitution (Figure S1) and of the nucleotide gradient across the liposomal membrane (Figure 4). Additional analyses confirmed that also the position of the His tag (whether C-terminal or N-terminal) did not affect the observed substrate preference pattern (Figure S2).

The determination of the kinetic parameters revealed higher affinities of reconstituted *Pam*NTT1 for both adenine nucleotides when ADP, instead of ATP, was present at the proteoliposomal interior (Table 2). Moreover, *Pam*NTT1 generally exhibited higher maximal velocities for ATP than for ADP import, unaffected by the type of exported substrate (Table 2). Influence of the exported substrates on nucleotide import was investigated in more detail. Interestingly, vesicles loaded with increased ADP concentrations in the absence of ATP or in the presence of the nonsubstrate GTP led to higher import rates when compared with the corresponding transport into liposomes loaded with both substrates ATP and ADP (Figure 3). Furthermore, increase of the ADP concentration and simultaneous decrease of the ATP concentration in the proteoliposomes resulted in intermediate kinetic parameters and shifted the low affinities for nucleotide import in counter-exchange with ATP towards the high import affinities of the nucleotide uptake into ADP-loaded vesicles (Table 2). Thus, decrease of nucleotide import in the presence of rising internal ATP/ADP ratios (Table 1) is a consequence of the reduction of the import affinities by the exported substrate ATP (Table 2). Because *Pam*NTT1-mediated nucleotide transport into liposomes loaded with decreasing ADP concentrations in absence of ATP was always substantially higher than the corresponding transport in presence of rising concentrations of the additional interior substrate ATP, a possible limitation of the counter-exchange substrate ADP (from 10 mM up to about 2.5 mM) was ruled out (Figure 3).

The stimulation of nucleotide import affinities by rising ADP/ATP ratios is in agreement with the physiological function of the carrier. A low-energy state of the bacterium (simulated by a high ADP/ATP ratio in the liposome) would consequently promote total nucleotide import, and would additionally stimulate ATP import to a higher extent than ADP import. Since not only the bacterial ATP level is subject to fluctuations, the effect of different external ADP/ATP ratios on nucleotide uptake was analyzed to mimic different energy states of the host cell (Figure 6). The determined competition pattern indicates that not even high concentrations of ADP could markedly affect ATP import into ATP-loaded proteoliposomes (Figure 6D), whereas ADP import into ATP-loaded proteoliposomes was already exceedingly suppressed by low concentrations of ATP (Figure 6A). The biochemical characteristics of the reconstituted *Pam*NTT1 facilitate an effective accumulation of ATP in the liposome—even in the presence of exceeding amounts of exterior ADP (Figure 6)—and in particular when the internal ATP/ADP ratio is low (Tables 1 and 2).

These results confirm and mechanistically explain the postulated physiological role of this carrier, and at the first glance not only argue for only one mode of orientation in the liposomal membrane but also for a directed insertion, which corresponds to the native state.

One important step towards the characterization of reconstituted proteins is the identification of their orientation in the vesicle. However, directionality of protein insertion varies with the method of reconstitution used (for review, see [40–42]). Orientation analyses of *Pam*NTT1 showed that this protein in the applied system consistently exhibits a bidirectional, nearly 1:1 orientation in the lipid vesicle (Figure 7). An extended analysis by the combination of endoproteases Asp-N and factor Xa, accompanied by additional transport measurements, revealed that only the fraction of *Pam*NTT1 orientated with the N-terminally located His tag towards the liposomal lumen is functional (Figure 8 and 9). In this fraction of *Pam*NTT1, the exterior Asp-N cleavage sites are obviously not accessible (Figure 10). On closer inspection, a small fraction of *Pam*NTT1 that was directed with the N terminus towards the interior also seems to have been digested by Asp-N. Since this cleavage has no influence on the transport activity, we assume that some of the carriers are correctly oriented but not functionally inserted. The calculated molecular weight of the largest fragment resulting from Asp-N cleavage of *Pam*NTT1 inserted with the His tag to the exterior is 27 kDa. The complete digestion of *Pam*NTT1 exposing the His tag to the exterior into fragments substantially smaller than 27 kDa argues for the accessibility of additional cleavage sites and leads to the assumption that a significant fraction of *Pam*NTT1 is not correctly (completely) inserted. Because the calculated specific activity refers to the total protein applied for reconstitution, the high activity of reconstituted *Pam*NTT1 is even underestimated.

Additionally, in the applied liposomal system, the orientation of ADP/ATP carriers from yeast mitochondria was analyzed by the help of the specific inhibitors bongkrekic acid (BKA) and carboxyatractyloside (CAT). AAC-mediated transport was strongly inhibited by 10 μM CAT (to 52%) or by 10 μM BKA (to 20%) when used separately, and was completely inhibited (to about 0.5%) when both inhibitors where applied simultaneously (unpublished data). This reveals that the reconstituted mitochondrial AACs exhibited a nearly 1:1 orientation and that the right-side-out as well as the inside-out oriented carriers were functional under conditions used for the orientation analyses of the reconstituted *Pam*NTT1.

Until now, the sense of integration of the *Pam*NTT1 in P. amoebophila has been unknown, and therefore, a comparison of the orientation of the reconstituted functional protein with the native state was impossible. However, an analysis of the topology of proteins in membranes revealed that most of them exhibit an accumulation of positively charged amino acids at the cytosolic side [43]. In compliance with the predicted topology (Figure 10), the hydrophilic loops connecting the transmembrane domains 6, 7, 10, and 11 of *Pam*NTT1 contain a remarkable accumulation of positively charged amino acids, and therefore these positively residues are probably exposed to the cytosol of P. amoebophila. The demonstrated orientation of the functional fraction of *Pam*NTT1 in the lipid vesicles is consistent with the predicted orientation of the native transporter. Whether *Pam*NTT1 exhibits 11 (as given in the current model) or 12 transmembrane-spanning helices (as previously described for structurally related NTTs) will be analyzed in future [17,18,29].

Despite the fact that the biochemical properties of the reconstituted *Pam*NTT1 are in line with the physiological function of this carrier in *P. amoebophila,* the obtained results are surprising. Given that at a pH 7.5, ATP and ADP exist mainly as the species ATP^4−^ and ADP^3−^_,_ a 1:1 stoichiometry of the hetero-exchange would consequently generate a charge difference across the membrane of the vesicle.

The electrogenic ADP/ATP exchange across the mitochondrial membrane was shown to be regulated by the energy state of the organelle. In energized mitochondria, the gradient of the substrate concentration across the inner mitochondrial membrane as well as the membrane potential (positive outside) supports the import of ADP^3−^ in exchange with ATP^4−^_,_ whereas the absence of a membrane potential results in similar transport rates for both adenine nucleotides (Figure 5) [34]. In contrast to the mitochondrial adenine nucleotide exchange, NTT-mediated transport seems to be unaffected by the membrane potential and therefore electroneutral (Figure 5).

The parasitic protist Entamoeba histolytica has reduced mitochondria homologs that lack an electron transport chain. The mitosomal AAC was shown to facilitate an electroneutral ADP/ATP exchange possibly by the uptake of a positive counter ion [44].

Isolated Rickettsia prowazekii cells prefer ATP instead of ADP import in diverse buffer media lacking phosphate [45]. However, rising concentrations of phosphate stimulated ADP import. Because rickettsiae are able to generate ATP through oxidative phosphorylation, the capacity to import ADP in the presence of phosphate is disadvantageous in a host cell that suffers from the infection [45]. The reduced expression of the ATP/ADP translocase gene in heavily infected host cells could prevent the loss of rickettsial ATP in exchange for host-derived ADP [46,47].

Until now, it has not been clear whether *Pam*NTT1 compensates the charge difference occurring due to ATP/ADP exchange by the transport of a counter ion. However, since the buffer media used for reconstitution and uptake measurements contained no phosphate (apart from possible traces by contamination), transport of ADP and phosphate in exchange with ATP is unlikely.

Our studies revealed that the biochemical characteristics of the reconstituted *Pam*NTT1 differ in some aspects from the results obtained with the E. coli system expressing *Pam*NTT1 and homologous NTT proteins [18]. It is known that E. coli expressing recombinant carrier proteins suffer and exhibit a comparably high cytosolic ADP/ATP ratio [48]. In the proteoliposomal system, such conditions favour ATP import (Figure 1, Tables 1 and 2), whereas E. coli exhibits nearly identical apparent affinities for both adenylates in the presence [18] and the absence of phosphate (unpublished data). Thus, in contrast to the ATP/ADP transporter from *R. prowazeckii, Pam*NTT1-mediated ADP import seems to be unaffected by phosphate [45].

Analysis of the substrate spectrum revealed that *Pam*NTT1 is highly specific for ATP and ADP, independent of the applied system (unpublished data and [18]). At this stage, it is not possible to decide which system is closer to the native state, because the E. coli system as well as proteoliposomes are in vitro models. E. coli differs considerably in form, size, and lipid composition from liposomes, and these differences might be the cause of the observed alterations in the biochemical properties of *Pam*NTT1.

It is remarkable that the recombinant *Pam*NTT1 exhibits a “sided-ness,” which allows an efficient import of ATP in exchange with ADP when reconstituted into liposomes but not when measured in E. coli, in particular since the E. coli system at a first glance seems to be closer to the native environment. In fact, some points could also argue for the liposomal system as a better model for studying *Pam*NTT1. First of all, both *Pam*NTT1 proteoliposomes and P. amoebophila are smaller than E. coli cells [18,38], and therefore, an influence of the membrane curvature on *Pam*NTT1 function can also be assumed. Second, it was shown that trafficking of host-derived phospholipids to C. trachomatis occurs, and thus the intracellular bacterium in contrast to E. coli harbors (about 40%) eukaryotic phosphatidylcholine (PC) in the plasma membrane [49–51]. Whether there is a possible correlation between the membrane curvature or the phospholipid PC and the functionality (in particular the “sided-ness”) of *Pam*NTT1-mediated transport is the topic of our future studies.

Taken together, heterologous expression of nucleotide transport proteins in E. coli is clearly suitable in identifying basic functional properties and mode of transport of these proteins, whereas the complexity of the E. coli cell prevents a more detailed functional analysis, which becomes feasible in the liposomal system.

We suggest that the sophisticated, stimulating effect of the exported substrate ADP on import affinities of *Pam*NTT1, which was observed (under diverse conditions) in the less-complex liposomal system (Figures 2 4, S1, and S2 and Tables 1 and 2), is an intrinsic characteristic of the native *Pam*NTT1. This characteristic allows energy import into a bacterial cell that exhibits a reduced energy state even under conditions of a high ADP/ATP ratio in the host cell. Therefore, it also becomes evident why bacterial ATP/ADP transport proteins fundamentally differ in structure from mitochondrial AACs, which mediate energy export [31–34,52].

Interestingly, an influence of the exported substrate on import characteristics of an NTT protein has been previously observed on intact E. coli cells. The recombinant NAD^+^/ADP transporter from P. amoebophila (*Pam*NTT4) mediated NAD^+^ import in counter-exchange with ADP and also ADP and NAD^+^ homo-exchange, respectively, whereas the physiologically unfavourable import of ADP in counter-exchange with NAD^+^ was nearly unobservable [16]. Whether the observed biochemical characteristics of *Pam*NTT4 are also caused by different kinetic parameters at interior and exterior and by a stimulating influence of the exported substrate ADP on import affinities and whether the trans-stimulating effect of ADP is a common feature of all bacterial and plastidic ATP/ADP transporters remains to be investigated.

The establishment of an expression, purification, and reconstitution protocol for a representative NTT from intracellular living bacteria and the generated *Pam*NTT1-specific antibodies are fundamental prerequisites for crystallization; detailed structure–function analyses in the future and will also provide important guidance for NTT inhibitors, which might represent suitable drugs for specific anti-chlamydial therapy.

## Materials and Methods

### Heterologous synthesis of *Pam*NTT1 and purification of E. coli membranes.

To generate C-terminal His tag fusion constructs, the *Pam*NTT1 coding sequence was amplified from the pET 16b construct [18] by PCR using Pfu polymerase and the following oligonucleotides: NTTBamHI_sense 5′-GGATCCATGTCGCAAGATGCGAAACAGAC-3′, NTTNheI_sense 5′-GCTAGCATGTCGCAAGATGCGAAACAAGAC-3′, and NTTXhoI_antisense 5′-CTCGAGGCTAGTAGCTATTTCCGATGT-3′. The PCR products containing the generated NheI/XhoI or BamHI/XhoI sites were inserted into a pET 21a plasmid (Merck Biosciences; http://www.merckbiosciences.co.uk/home.asp). Dependent on the position of the insertion in the pET 21a plasmid, the resulting proteins were extended with either a C-terminal His tag (NheI/XhoI) or a C-terminal His tag and an N-terminal T7 tag (BamHI/XhoI). DNA manipulations were performed essentially as described in Sambrook et al. [53]. Correctness of the constructs was proven by complete sequencing (NanoBioCenter, TU Kaiserslautern, Germany).

The pET16b as well as the pET21a constructs containing *Pam*ntt1 were transformed into BLR(DE3)pLysS cells (Merck Biosciences). The transformed bacterial cells were grown at 37 °C in Terrific Broth medium [48]. At an A_600_ of 0.5–0.6 initiation of T7 RNA polymerase expression was induced by addition of isopropyl-β-d-thiogalactopyranoside (1 mM). The cells were harvested 4 h post-induction by centrifugation (5,000*g*, 10 min, 20 °C), resuspended in buffer medium (1 mM EDTA, 15% glycerol, 10 mM Tris, pH 7.0) and immediately frozen in liquid N_2_. To induce autolysis by the endogenous lysozyme, frozen cells were thawed at 37 °C and treated with a pinch of DNAse and RNAse. To avoid proteolytic activity, phenylmethanesulphonylfluoride (PMSF) (1 mM) was added. Cell lysis was supported by sonication. In the first centrifugation step (20,000*g*, 15 min, 4 °C), cell debris were removed and membranes were collected from the supernatant by centrifugation (100,000*g*, 30 min, 4 °C).

### Purification of *Pam*NTT1 by IMAC.

All purification steps were carried out at 6 °C. Membranes (see above) were resuspended in buffer medium B (10 mM NaCl, 5 mM imidazole, 50 mM Na_2_HPO_4_, pH 7.9) containing 1% DDM (Glycon Biomedicals; http://www.glycon.de/), 70 mM NLS (Sigma; http://www.sigmaaldrich.com) or 1% Triton (Sigma) and stirred for 1 h to allow solubilization of intrinsic proteins. After centrifugation of the solubilized membranes (100,000*g*, 30 min, 4 °C), the supernatant was incubated with Ni-NTA agarose (Qiagen; http://www.quiagen.com) for 2 h. The Ni-NTA suspension was transferred onto a chromatography column, washed with 10 volumes of the Ni-NTA agarose bed volume with buffer medium B containing 0.5% DDM, 70 mM NLS, or 0.5 % Triton, and afterwards, with 6 column volumes of buffer medium W (10 mM NaCl, 60 mM imidazole, 50 mM Na_2_HPO_4_, pH 7.9, 0.5% DDM, 70 mM NLS, or 0.5% Triton). Subsequently, recombinant *Pam*NTT1 was eluted with an appropriate amount of buffer medium E (10 mM NaCl, 500 mM imidazole, 50 mM Na_2_HPO_4_, pH 7.9, 0.1% DDM, 35 mM NLS. or 0.2% Triton) to obtain a protein concentration of 0.4–0.5 mg/ml.

### Protein quantification.

Protein concentrations of the DDM-purified NTT were quantified according to the method described by Bradford [54]. 500 μl of Bradford solution were mixed with H_2_O and eluted protein (5–10 μl) to reach a final volume of 1000 μl. The same volumes of elution buffer (containing detergent) were used for the blank values. Protein concentrations of the NLS and the Triton-purified proteins were quantified photometrically at 280 nm. To reduce nonprotein absorbance generated by the high imidazol concentration in the elution buffer, the protein was desalted on a NAP-5 column equilibrated with buffer (10 mM NaCl, 50 mM Na_2_HPO_4_, pH 7.9, 35 mM NLS, or 0.2% Triton). The same buffer media were used for the blank values. All measurements were carried out in a BioPhotometer (Eppendorf; http://www.eppendorf.com).

### Preparation of proteoliposomes.

Immediately after purification of PamNTT1 (see above), 50 μl of the eluate was mixed with a homogeneous emulsion of 400 μl l-α-phosphatidylcholine (125 mg/ml, type IV-S, Sigma) in buffer medium TG1 (100 mM tricine, 30 mM potassium gluconate, pH 7.5). For loading, 50 μl of the indicated nucleotides were added to achieve the indicated concentrations. After vigorous vortexing, the mixture (500 μl) was frozen in liquid N_2_. Proteoliposomes were thawed on ice and sonified for 20 s (Sonifier 250, Branson; http://www.bransonultrasonics.com) at lowest output level and 50% duty cycle. Proteoliposomes (500 μl) were applied to a NAP-5 column (GE Healthcare; http://www.gehealthcare.com) equilibrated with TG2 (10 mM Tricine, 150 mM potassium gluconate, pH 7.5) to remove external nucleotides. Proteoliposomes were eluted from the column with 1000 μl of TG2. For protease cleavage assays, the NAP-5 column was equilibrated with cleavage buffer medium (100 mM NaCl, 20 mM Tris, 2 mM CaCl_2_, pH 8.0).

### Protease cleavage assays.

The endoproteases factor Xa (New England Biolabs; http://www.neb.com) and Asp-N (Sigma) were added to proteoliposomes to obtain a protein/protease ratio of 20/1. To allow a combination of Asp-N treatment and uptake experiments, the cleavage was carried out in buffer medium TG2. Protease activity was stopped with 1 mM PMSF (factor Xa) or 10 mM EDTA (Asp-N) at the indicated time points. All protease assays were carried out at 25 °C.

### Uptake of radioactively labelled ATP and ADP into proteoliposomes.

Radioactively labelled [α^32^P] ADP was synthesized enzymatically by using hexokinase following a standard protocol [55]. Quality and purity of synthesized [α^32^P] ADP was analyzed by thin layer chromatography as described elsewhere [55,56]. Proteoliposomes were added to 100 μl of TG2 containing [α^32^P] ATP (NEN; http://www.perkinelmer.com) or [α^32^P] ADP at indicated concentrations. For transport measurements, proteoliposomes were incubated at 30 °C and uptake was terminated at the indicated time periods by removal of residual nucleotides using anion exchange chromatography (Dowex 1 × 8 Cl, 200–400 mesh, Sigma) [34]. Liposomes were eluted from the chromatography column by addition of 1500 μl Tricine (200 mM, pH 7.5). Radioactivity in the eluate was quantified using a scintillation counter (Canberra-Packard; http://www.canberra.com).

### SDS-PAGE and immunostaining.

Before separation by SDS-PAGE, proteoliposomes were treated with SDS (4%) and with an appropriate volume of 6 × concentrated sample buffer medium (375 mM Tris/HCl, pH 6.8, 0.3% SDS, 60% glycerol, 1.5% bromophenol blue). Proteins were separated in a discontinuous, denaturing system with a 3% stacking and a 12% separating polyacrylamide gel as described by Laemmli [57]. Following electrophoresis, gels were stained [57,58] or proteins were transferred to a nitrocellulose membrane in a wet-blotting apparatus. Immunodetection was performed using a monoclonal anti poly His immunoglobulin G (IgG) (Sigma) combined with a secondary alkaline phosphatase conjugated anti-mouse IgG (Sigma), or by an anti-*Pam*NTT1–specific serum combined with a secondary alkaline phosphatase conjugated anti-rabbit IgG (Promega; http://www.promega.com). After staining with nitro blue tetrazolium chloride/5-bromo-4-chloro-3′-indolyl phosphate toluidine, salt blots were rinsed in water and air-dried. To estimate molecular masses, a prestained broad range protein marker (6–175 kDa, New England Biolabs), was used.

### Production of *Pam*NTT1-specific anti serum.

*Pam*NTT1-specific anti serum was generated at Eurogentec (http://www.eurogentec.com) by immunization of rabbits with IMAC-purified recombinant protein (see above).

## Supporting Information

Figure S1 Influence of the Detergent Used During Purification and Reconstitution on *Pam*NTT1-Mediated Nucleotide Uptake *Pam*NTT1 was solubilized, purified, and reconstituted in presence of the detergents Triton, NLS, or DDM. Proteoliposomes loaded with 10 mM of indicated counter-exchange substrate were incubated with 50 μM [α^32^P] labelled ADP or ATP. Nucleotide import was stopped in the time-linear phase by separation of external nucleotides from proteoliposomes by anion exchange chromatography. ATP uptake into ADP-loaded liposomes was set to 100% and the remaining exchanges were calculated accordingly. *Pam*NTT1 mediated ATP_im_/ADP_ex_ transport is given in nmol (mg protein)^−1^ h^−1^ above the corresponding white bar. ATP_im_/ATP_ex_ (light gray bars), ADP_im_/ADP_ex_ (gray bars), ADP_im_/ATP_ex_ (dark gray bars). The given data represent the net nucleotide import calculated by subtraction of the control (uptake into unloaded proteoliposomes).
(34 KB PDF)

Figure S2 Influence of the His Tag Position on *Pam*NTT1-Mediated Nucleotide Uptake Recombinant *Pam*NTT1 extended by an N-terminal His tag (N-His), a C-terminal His tag (C-His), or an N-terminal T7 tag and a C-terminal His tag (N-T7 + C-His) were solubilized, purified, and reconstituted in the presence of DDM. Proteoliposomes loaded with 10 mM of indicated counter-exchange substrate were incubated with 50 μM [α^32^P] labelled ADP or ATP. Nucleotide import was stopped in the time-linear phase of uptake by separation of external nucleotides from proteoliposomes by anion exchange chromatography.
(A) ATP uptake into ADP-loaded liposomes was set to 100% and the remaining exchanges were calculated accordingly. *Pam*NTT1-mediated ATP_im_/ADP_ex_ transport is given in nmol (mg protein)^ −1^ h^−1^ above the corresponding white bar. ATP_im_/ATP_ex_ (light grey bars), ADP_im_/ADP_ex_ (grey bars), ADP_im_/ATP_ex_ (dark grey bars). The given data represent the net nucleotide import calculated by subtraction of the control (uptake into unloaded proteoliposomes).
(B) Coomassie stained SDS-PAGE of IMAC purified, recombinant *Pam*NTT1. M, Molecular mass marker.
(253 KB PDF)

### Accession Number

The National Center for Biotechnology Information (http://www.ncbi.nlm.neh.gov) accession number for *Pam*NTT1 is AJ582021.

## Abbreviations

AAC: ADP/ATP carrier
DDM: *N*-dodecyl-β-maltoside
IMAC: immobilized metal affinity chromatography
NLS: *N*-lauroylsarcosine
NTT: nucleotide transporter
*Pam*: Protochlamydia amoebophila
Triton: Triton-X-100
