# Supplementary material for: Enlightening Energy Parasitism by Analysis of an ATP/ADP Transporter from Chlamydiae
Source: PLoS Biol. 2007 Aug 28;5(9):e231. doi: 10.1371/journal.pbio.0050231 (PMC1951785; doi:10.1371/journal.pbio.0050231)
Supplement: Supplementary file 1 [file pbio.0050231.sg001.pdf]

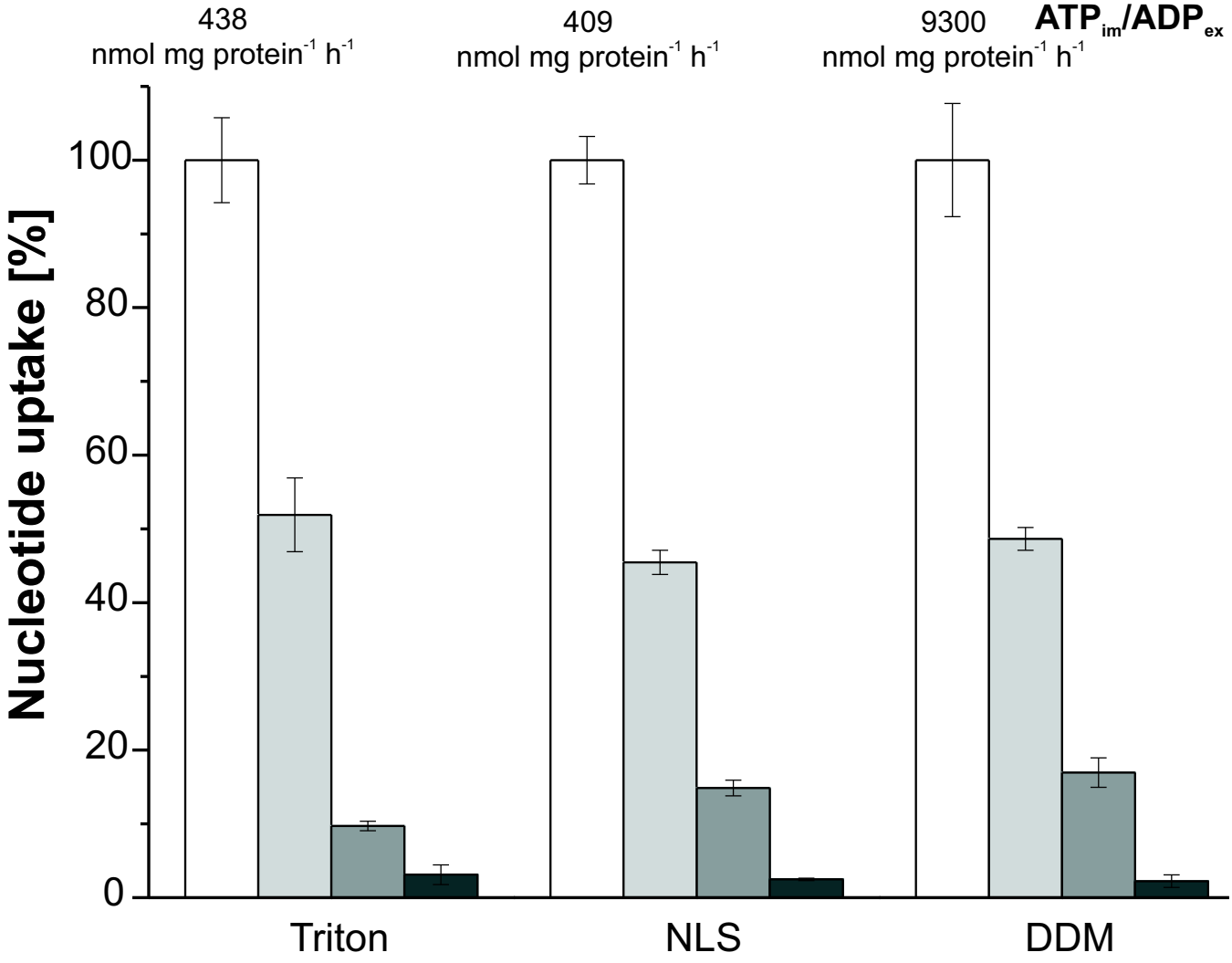

Supplement Figure 1.
